# Unseen intimate partner violence: regional differences in controlling behaviours

**DOI:** 10.3389/fpsyt.2026.1837243

**Published:** 2026-05-22

**Authors:** Enzel Özgenç Osmanoğlu, Ömer Alkan

**Affiliations:** 1 Department of Econometrics, Faculty of Economics and Administrative Sciences, Ataturk University, Erzurum, Türkiye; 2 Master Research, Training and Consulting Services Ltd. Co., Erzurum, Türkiye

**Keywords:** binary logistic regression, controlling behaviours, emotional violence, regional differences, Türkiye

## Abstract

**Introduction:**

Controlling behaviour is considered a form of emotional abuse and restricts an individual’s autonomy. This study aims to determine the factors associated with women’s exposure to controlling behaviour by their spouse/partner in the last year, as of the time the survey was conducted, by place of residence in Türkiye.

**Methods:**

This study utilised microdata obtained from the National Research on Domestic Violence against Women in Türkiye. Binary logistic regression analysis was employed to determine the factors associated with women’s exposure to controlling behaviour by their spouse/partner.

**Results:**

The results of the study showed that age, educational level, the woman’s greater contribution to household income, suicidal thoughts, previous pregnancies, the educational level of the spouse/partner, whether or not the man beating his wife is considered acceptable, the educational level of the spouse/partner, fear of the spouse/partner, the spouse/partner fighting with the man in a way that involves physical violence, the spouse/partner experiencing physical violence from their own family, exposure to other types of violence by the spouse/partner, knowledge of preventive measures against violence, and household size were found to be associated with exposure to controlling behaviour.

**Discussion:**

The study found that the significance and effect of variables related to exposure to controlling behaviour also differed by women’s place of residence. According to the study, appropriate strategies to reduce controlling behaviour should be developed, taking into account public health programmes to raise awareness of controlling behaviour towards women, based on their place of residence, and considering the determinants that influence exposure to controlling behaviour.

## 1 Introduction

Violence against women is one of the most widespread and systematic forms of gender-based discrimination worldwide. This type of violence is not limited to physical or sexual assaults; it also includes controlling behaviours that restrict a woman’s rights to make decisions, move freely, and participate in social life. In the literature, such behaviours are generally examined under the concept of ‘coercive controlling’ and encompass emotional, economic and psychological forms of pressure (1). Intimate partner violence (IPV) is one of the most common forms of violence against women, including physical, sexual, psychological and controlling behaviour perpetrated by a male partner or former partner. This type of violence is a significant public health issue and constitutes a violation of women’s human rights (2). At the same time, IPV clearly highlights the inequality between men and women (3). Physical violence encompasses actions such as hitting, pushing, slapping, or other acts aimed at causing bodily harm to an individual, and is one of the most visible types of violence (4). Psychological/emotional violence includes behaviours that damage an individual’s self-esteem and mental well-being, such as humiliation, threats, constant criticism, intimidation, and social isolation. It is often considered a precursor to physical violence (1). Sexual violence is defined as forcing or subjecting an individual to sexual acts without their explicit consent, or using sexuality as a tool of coercion and control. This includes not only physical coercion but also situations where consent is eliminated through threats, manipulation, pressure, or an imbalance of power (5). Economic violence refers to making an individual dependent by restricting their access to economic resources, controlling their income, preventing their participation in the workforce, or systematically limiting their financial independence (5, 6). While there are no sharp boundaries between these types of violence, it is clear that there is a dynamic relationship in which they reinforce each other.

Controlling behaviours are defined as an individual attempting to mould their partner according to their own desires, values, and expectations, interfering in their partner’s decisions by restricting their social relationships, interfering with their clothing style, monitoring their digital and physical activities, and hindering their economic independence. This can be considered a component of psychological violence (7). Controlling behaviours are often not as visible and measurable as physical violence, resulting in a low level of social awareness. Social norms frequently legitimise these behaviours on grounds such as jealousy or protection, and may even be internalised by the women who are subjected to them (8, 9). All directives regarding who your partner may meet with, how they should dress, when they should return home, how they should use social media, whether they should work, or to what extent they should communicate with their family are evaluated within this scope. Controlling behaviours are closely related to other forms of violence and play a role in structuring the continuity of violence (1, 10, 11). In this context, it can be said that different forms of violence have a mutually reinforcing relationship and that controlling behaviours, in particular, play a central role within this structure, supporting the emergence and continuity of other types of violence.

Factors such as women’s economic and social empowerment, income generation, increased education levels, and widespread access to social networks can alter the power balance in relationships and reduce the risk of violence by limiting a partner’s controlling behaviour (12, 13). It is hypothesised that women’s increasing economic independence can make it more difficult for partners to maintain coercive control strategies such as economic restriction, isolation, and limited freedom of movement. However, the literature also emphasises that this relationship does not always function protectively. Particularly in societies with high gender inequality, women’s empowerment can be perceived as a threat to traditional gender roles, leading to a “backlash” effect and an increase in controlling and violent behaviour by the partner (14, 15). Therefore, it can be argued that the relationship between women’s empowerment and their partners’ controlling behaviour is shaped by gender norms, structural inequalities, and intra-relationship power dynamics rather than individual factors (1, 16).

Approximately 30 per cent of women worldwide (roughly one in three) experience physical, emotional, psychological or sexual violence from their partners. Controlling behaviour is seen as a component of emotional violence within intimate partner violence (2). The rate varies by country and region. According to OECD (2023) data, approximately 32 per cent of women aged 15–49 in Türkiye have experienced physical and/or sexual violence from their current or former partners, and this rate is quite high amongst OECD countries (17). It has been found that a significant proportion of women are controlled by their partners in many areas, from their physical mobility to their social relationships (18). These data indicate that controlling behaviour is not only an individual issue, but also a cultural and structural one.

While physical violence, a type of intimate partner violence, is significantly affected by potential changes in societal norms and policies, psychological/emotional violence, or controlling behaviours perpetrated against women by their partners, can be considered more resistant to such changes because they are more difficult to measure and address (19, 20). In the specific case of Türkiye, the compulsory education policy implemented in 1997 has been shown not to have affected psychological violence perpetrated against women by their partners (21). Therefore, it is clear that controlling behaviours, considered a sub-component of psychological violence, will likely follow the same trend.

In the literature, it is reported that partners’ controlling behaviour negatively affects not only psychological well-being but also women’s participation in social circles, economic independence, and decision-making capacity (11, 22). It is also emphasised that such behaviour can pave the way for more severe forms of violence, such as physical and sexual violence, over time (23). The frequency with which women are subjected to these control mechanisms, and how they perceive these behaviours, vary across cultural contexts and gender norms. In societies where patriarchal structures are more dominant, such behaviours are more prevalent; controlling women is seen as part of the norms of masculinity (24–26). However, the extent to which women are subjected to controlling behaviour by their partners varies from person to person, culture to culture and region to region, and this must be taken into account in the development of policies to combat violence (18). For example, whilst raising awareness and education programmes may be a priority in rural areas, economic support and advisory services may be more effective in large cities. Such regional approaches will enable efficient resource use and targeted interventions.

Different regions of Türkiye exhibit diversity in terms of cultural factors such as patriarchal norms, concepts of honour, and gender relations. In this context, the frequency and form of controlling behaviour towards women also show regional differences. The rate of women being controlled by their partners is higher in the Eastern and Southeastern Anatolia regions compared to the Western and Marmara regions. The stricter nature of gender norms in rural settlements, the prominence of patriarchal family structures, and the low level of economic independence among women play a significant role in this difference (26). Analysing these norms on a regional basis provides a better understanding of the root causes of violence (27).

In this context, it is understood that forms of partner control in Türkiye vary not only at the individual level but also across regions, depending on cultural and structural factors. However, the existing literature contains very few studies that systematically address these regional differences in the Turkish context. Examining not only the existence of controlling behaviour towards women, but also its regional distribution and the socio-cultural reasons for this difference, will fill an important gap in this field.

This study takes a multidimensional approach to investigating regional differences in controlling behaviour towards women by their partners in Türkiye; the social context of this behaviour, its individual and structural causes, its effects on women, and intervention approaches are evaluated in light of the literature. To our knowledge, this study is the first to identify factors associated with women’s exposure to controlling behaviour by their partners according to regional differences in Türkiye. Thus, it aims to draw attention to this less visible but equally destructive form of violence against women and to raise social awareness on this issue.

This study is grounded in the framework of coercive control and gendered power relations within intimate partnerships. Coercive control refers to a pattern of behaviours through which one partner seeks to dominate, regulate, and restrict the autonomy of the other, often operating through non-physical forms of violence such as emotional manipulation, surveillance, and social isolation. From this perspective, controlling behaviours are not isolated acts but part of a broader system of power asymmetry shaped by socio-economic conditions, gender norms, and intra-household dynamics.

In addition, this study draws on the women’s empowerment framework, which suggests that increases in women’s education, economic participation, and decision-making power may alter traditional gender hierarchies. While empowerment may reduce exposure to violence in some contexts, it may also trigger a “backlash effect,” whereby male partners intensify controlling behaviours in response to perceived threats to their authority. Therefore, the selection of independent variables in this study is guided by their relevance to these theoretical mechanisms, including individual characteristics, partner attributes, and relational and structural factors that shape power dynamics within intimate relationships.

## 2 Literature review

Violence against women is recognised as a global health issue (28). Partner violence against women is not limited to physical or sexual violence, but is a multidimensional phenomenon that also includes emotional, psychological and, in particular, controlling behaviour. Controlling behaviour manifests itself as restricting, monitoring, directing, or limiting one’s partner’s decisions or personal space (29). These behaviours are often normalised by both the perpetrator and the victim, justified under the guise of proving love, jealousy, possessiveness or protection (30). Patriarchal social structures and gender norms normalise such behaviour by presenting men’s control over women as ‘establishing order within the family’ or ‘the duty of manhood’ (31). Furthermore, romantic love myths and social expectations can lead young women in particular to interpret their partners’ jealousy and control as a sign of affection. In this context, the literature on how controlling behaviour directed at women by their partners is addressed at both the individual and structural levels is critical to understanding the invisible forms of violence against women.

Studies examining women’s exposure to controlling behaviour by their partners show that 35% of women in European Union countries report having been subjected to controlling behaviour by their partners at least once in their lives (2). In Türkiye, 81.7 per cent of women stated that they had been subjected to controlling behaviour by their partners at least once in their lives (18). In Sweden, the corresponding rate is 28 per cent (32). In rural areas of South Africa, it has been found that 55.8 per cent of women have experienced controlling behaviour at least once in their lives (33). In England and Wales, it is a criminal offence for women to be subjected to controlling behaviour by their partners (34). In India, approximately 48 per cent of married women experience controlling behaviour from their partners, whilst 31 per cent experience violence from their partners (35, 36). In the Philippines, 9% of women stated that there were justifiable reasons for their husbands to abuse them physically.

In comparison, 18% stated that they had been subjected to intimate partner violence at least once in their lives. 36% of women stated that they had been subjected to controlling behaviour by their husbands (37). In Rwanda, 29.2%, 22.2% and 12.4% of women reported experiencing physical, psychological and sexual intimate partner violence, respectively, whilst 52.1% reported experiencing controlling behaviour by their partners (38).

Women’s exposure to controlling behaviour by their partners is related to certain key variables. The likelihood of exposure to intimate partner violence decreases with increasing age, higher levels of education, and greater contribution to household income (18, 39). In addition to these variables, women living in rural areas are more exposed to intimate partner violence than women living in cities (18, 39, 40). Again, contrary to these studies, another study found a correlation between place of residence and women’s exposure to intimate partner violence (37). Compared to married women, single women, women whose native language is Turkish, women exposed to poor or very poor health conditions, and women who fear their intimate partners are more likely to experience intimate partner violence (18, 41). In addition to these studies, some studies have also revealed that, in addition to demographic variables such as educational level and marital status, variables such as witnessing the father’s abusive behaviour, being a victim of domestic violence, and being aware of the law on violence against women are associated with women being subjected to controlling behaviour by their partners (24, 37, 42).

Another important variable associated with women being subjected to controlling behaviour by their partners is the legitimisation of controlling behaviour by linking it to a justifiable reason (18, 24, 36, 38). Some women initially interpret their partners’ controlling behaviour as ‘interest’, “jealousy” or ‘a sign of love’ and therefore view such behaviour as legitimate. This situation is rationalised by associating it with romantic ideals in an emotional context (30). Another study uses the concept of ‘intimate terrorism’ to describe a partner’s systematic attempts at control. It states that such controlling behaviour is legitimised under the pretext of ownership or protection. Controlling behaviour towards women can be concealed under the guise of love and devotion (29). Similarly, another study states that men often justify their control over women by invoking patriarchal gender norms such as ‘authority,’ ‘protecting the family,’ or ‘masculine responsibility’ (31). Another study shows that common sayings such as ‘If a man takes ownership, he becomes jealous’ legitimise controlling behaviour at a societal level (43). In the Turkish context, men’s controlling behaviour is legitimised within the framework of traditional values such as ‘honour’, ‘women knowing their place’ and ‘obedience’. These behaviours are sometimes justified by family responsibilities and norms of masculinity (44).

In addition to the factors listed above, other variables are associated with women being subjected to controlling behaviour by their partners. A significant relationship has been identified between women’s feminist identity and commitment to the relationship and their exposure to controlling behaviour by their partners (24). Another study found a significant correlation between belief in romantic relationships and women being subjected to controlling behaviour by their partners (45). It has been found that as women’s decision-making power increases, the likelihood of them being subjected to controlling behaviour by their partners decreases (36). In another study, no significant relationship was found between women’s decision-making power and their likelihood of being subjected to controlling behaviour by their partners (37). It has been found that women who experience economic, physical and emotional violence are more likely to be subjected to controlling behaviour by their partners (18). It has been found that in families where decisions are made jointly, women are less likely to be subjected to controlling behaviour by their partners. In contrast, when either the male or female partner makes decisions alone, women are more likely to be subjected to controlling behaviour by their partners (39).

Certain characteristics of partners (men) influence the extent to which women are subjected to controlling behaviour by their partners. It has been observed that as partners’ levels of education increase, the likelihood of women being subjected to controlling behaviour by their partners decreases (37, 39). It has been found that a partner’s alcohol consumption contributes to an increase in physical and sexual intimate partner violence against women (46). In contrast to this study, another study found that partners’ alcohol consumption had no significant effect on women’s exposure to controlling behaviour by their partners (37). It has been observed that men who were sexually abused during childhood and who experience greater difficulty controlling their sexual urges are more likely to be subjected to controlling behaviour by their partners (46). In addition to these variables, it has been determined that partners’ adherence to patriarchal norms has a significant effect on women’s exposure to controlling behaviour by their partners (42). It has been found that partners who exhibit controlling behaviour in their own lives are more likely to engage in controlling behaviour towards their spouses (47, 48).

Controlling behaviour by partners towards women leads to various negative consequences, including physical intimate partner violence (47). However, it has been found that women who are subjected to controlling behaviour by their partners are inclined to physically punish their children (49).

## 3 Materials and methods

### 3.1 Data source

The National Research on Domestic Violence against Women in Türkiye is one of the most comprehensive studies conducted nationwide to understand the dimension, content, causes, effects and risk factors of domestic violence experienced by women in Türkiye. It was conducted for the first time in 2008 to determine the different aspects and reasons of violence against women and to meet the requirement of collecting data on this issue. The National Research on Domestic Violence against Women in Türkiye, conducted in 2014, is significant in terms of reflecting the change regarding violence against women since the research conducted in 2008 (50, 51).

The study utilised cross-sectional data from the National Research on Domestic Violence against Women in Türkiye conducted by the Population Studies Institute at Hacettepe University in 2014. The research questionnaire was designed by taking into account the questionnaire applied by the WHO in the study titled “Multi-country Study on Women’s Health and Domestic Violence against Women” (52). New questions regarding country-specific requirements, particularly targeting the monitoring of legal regulations, were also added to the questionnaire (50, 51).

The most recent National Research on Domestic Violence against Women in Türkiye data shared by Hacettepe University Institute of Population Studies is that of the year 2014. This research’s main strength and advantage is very significant, and unfortunately, the actual topic is always suicidality. A particularly important and neglected topic is women’s mental health. The sample size is more than enough, although the data is from 12 years ago.

The National Research on Domestic Violence against Women in Türkiye is one of the most comprehensive studies conducted nationwide to understand the dimension, content, causes, effects and risk factors of domestic violence experienced by women in Türkiye. It was initially displayed in 2008 to ascertain the various facets and causes of violence against women as well as to fulfil the need for data collection on this matter. In terms of illustrating the evolution of domestic violence against women since the 2008 research, the National Research on Domestic Violence against Women in Türkiye conducted in 2014 is noteworthy (50, 51).

Within the scope of the violence survey, Türkiye has been divided into 30 strata to enable estimates at the national, urban/rural, regional, and sub-regional levels. Except for the Istanbul region, one of the 12 regions, the other regions have been distributed between urban and rural strata at approximately 75% to 25%. In Istanbul, approximately 5% of households were selected from rural areas. In the study, settlements with a population of 10,000 or more constitute the urban stratum, whilst settlements with a population of less than 10,000 constitute the rural stratum. The study’s sample is a cluster sample (50, 51).

The fieldwork for the 2014 study commenced on 8 April 2014 and was completed on 11 July 2014. The research team administered the questionnaires for the National Research on Domestic Violence against Women in Türkiye. The WHO’s ethical guidelines were applied at every stage of the research, and several measures were taken to ensure the safety of both the women interviewed and the research team. Before each interview, respondents’ consent was obtained, and the interviewers signed the questionnaire to confirm it. The researchers were trained in ethical and safety rules and demonstrated sensitivity to the subject matter at the beginning of the interview, throughout the interview, and after the interview. In households with more than one woman in the 15–59 age group, a random method was used to select one woman so that the same questions were not asked to more than one woman in the household, and interviews were conducted with a single woman identified in each household. The research teams took great care to ensure that interviews with the selected women were conducted in a private setting. In addition, all women participating in the interviews were informed about the confidentiality of the interviews. During the consent and information processes, participants were also informed that their answers would be kept confidential (51).

In the 2014 survey, face-to-face interviews were conducted with 7,462 women aged 15–59 in 11,247 sample households. Although the questionnaires were completed, the refusal rate was 4.4%. The response rate for women’s interviews was 83.3% (51). Weight adjustments for women have been added to these datasets in accordance with the study’s sample design (51).

In the National Research on Domestic Violence against Women in Türkiye, questionnaires were administered in the form of a household questionnaire (asking about the number of individuals in the household, the number of rooms in the household, indicators of welfare level, etc.) and a women’s questionnaire (asking about the socio-demographic and other characteristics of the woman and her spouse/partner). The data in question was presented in two separate Excel files. The Excel files containing household and women’s data were combined into a single data file. After combining the files, 392 women whose household information could not be accessed were excluded from the analysis, leaving 7,070 women for analysis. As the study examined the exposure of married women, women who had been in a relationship, or women currently in a relationship to controlling behaviour by their spouse/partner, data from 612 women who had never been in a relationship at the time of the survey were excluded from the analysis. Consequently, the number of units to be considered was calculated as 6,458.

In addition, the survey’s conceptual foundation is broadly consistent with the ecological model of violence proposed by Heise (8), which conceptualises violence as a result of interacting factors at the individual, relational, community, and societal levels. The structure of the Turkish survey reflects this multidimensional perspective by incorporating variables related to individual characteristics, partner attributes, household context, and broader socio-cultural norms. This alignment strengthens the theoretical grounding of the data and supports the analytical framework adopted in this study.

### 3.2 Outcome variables

According to the National Research on Domestic Violence against Women in Türkiye, the women participating in the survey were asked the following questions regarding their exposure to controlling behaviour in the past year at the time the survey was conducted:

“Does/did your spouse or partner try to prevent you from seeing your friends?”

“Does/did your spouse or partner try to restrict/prevent you from seeing your family and close relatives?”

“Does/did your spouse or partner always want to know where you are?”

“Does/did your spouse or partner disregard you and behave indifferently towards you?”

“Did your spouse or partner get angry if you spoke to other men?”

“Did your spouse or partner suspect you of cheating on them?”

“Did your spouse or partner require your permission to visit any healthcare institution when you had a health problem?”

“Would your spouse or partner interfere with your clothing choices, demanding that you dress in a certain way?”

“Would your spouse or partner interfere with your use of social media sites such as Facebook or Twitter?”

The experiences of women exposed to controlling behaviour, as measured by the aforementioned questions, were considered to form the dependent variable. If the participating women had experienced at least one of the above situations in the last year at the time of the survey, it was assumed that they had been exposed to controlling behaviour by their spouse/partner; if they answered no to all of these items, it was assumed that they had not been exposed to controlling behaviour.

The dependent variable in the study is whether women were exposed to controlling behaviour in the year before the survey, by place of residence (urban or rural). Women who participated in the study and were exposed to controlling behaviour by their spouse/partner were coded as ‘1’, whilst those who were not exposed were coded as ‘0’. A separate binary logit model was established for both urban and rural areas in the study.

The construction of the dependent variable as a binary indicator based on multiple controlling behaviours is consistent with prior research on intimate partner violence, where composite measures are frequently used to capture complex and multidimensional phenomena. Although individual behaviours differ in severity and form, combining them into a single indicator enables the identification of exposure to controlling behaviour, which is theoretically meaningful within the coercive control framework. Nevertheless, it is acknowledged that this operationalisation may mask heterogeneity across different types of controlling behaviours.

### 3.3 Independent variables

The independent variables included in this study are derived from the National Research on Domestic Violence against Women in Türkiye. Each of these variables was identified through a literature review. Demographic and socio-economic variables related to women: generation (1946-1964 (Baby Boom Generation), 1965-1980 (Generation X), 1981-1995 (Generation Y), 1996 and later (Generation Z)) (53, 54), level of education (illiterate, primary school, middle school, high school, university) (54, 55), whether the woman contributes more to the household income (no, yes) (18), fear of spouse/partner (no, yes) (56), thoughts of suicide (no, yes) (57) and previous pregnancy (no, yes) (58), knowledge of measures to prevent violence (no, yes) (59) and household size (3 and under, 4-5, 6 and above) (60, 61) were determined.

Factors related to the woman’s spouse/partner: spouse/partner’s level of education (illiterate, primary school, secondary school, high school, university) (54), whether the spouse/partner has physically fought with the man (no, yes) (31), whether the man beating his wife is considered acceptable (no, yes) (62, 63), whether the spouse/partner has experienced physical violence from their family (no, yes) (63).

Other variables that may be related to violence: exposure to economic violence by spouse/partner (no, yes) (18, 64), exposure to emotional violence by spouse/partner (no, yes) (65, 66), exposure to physical violence by spouse/partner (no, yes) (65–67).

The theoretical framework informs the selection of independent variables of coercive control and gendered power relations. Specifically, variables related to women’s socio-demographic and economic characteristics capture dimensions of empowerment and autonomy, whilst partner-related variables reflect power asymmetries and behavioural tendencies within the relationship. Additionally, variables related to other forms of violence are included to account for the interconnected and mutually reinforcing nature of different types of intimate partner violence. This theoretically informed selection allows for a more comprehensive understanding of the mechanisms underlying controlling behaviours.

Although these variables are conceptually related, their simultaneous inclusion in the model is theoretically justified. Controlling behaviour represents an overarching pattern of domination, whilst emotional and physical violence capture specific manifestations of abuse, and fear reflects the psychological outcome of these dynamics. Including these variables together allows for a more comprehensive modelling of the relational context in which controlling behaviours occur, rather than treating them as isolated phenomena.

### 3.4 Analysis method

Survey statistics in Stata 15 (Stata Corporation) were used to account for the complex sampling design and weights. Weighted analysis was performed. First, the dependent variable, which was women’s exposure to controlling behaviour by their spouse/partner, and the frequencies and percentages of the independent variables were obtained. In this study, the binary logistic regression method was used to investigate differences in exposure to controlling behaviour according to place of residence.

The primary reason for the binary dependent variable in the binary logit model is that the model is designed to explain a probability-based decision-making process. This model was developed to examine how individuals arrive at a choice or probability of occurrence between two opposing situations (e.g., “yes/no”, “exposed/not exposed”). While classical linear regression models assume a continuous dependent variable, in the binary logit model, the dependent variable takes only two values, represented by the Bernoulli distribution. In this framework, the outcome for each observation is modelled as the probability 1 P(Y = 1|X) and linearised using a logit transformation. This approach guarantees that probabilities remain in the 0–1 range, eliminating the out-of-bounds estimation problem observed in linear regression (68, 69). This structure is widely used, particularly in the social sciences, health research, and economics literature, because many behavioural outcomes are inherently binary. Therefore, the binary logit model is considered a powerful econometric tool that both theoretically preserves probability limits and is suitable for explaining two-state decision processes (69, 70).

In addition to estimating separate models for urban and rural samples, an alternative empirical strategy could involve a pooled model with interaction terms between place of residence and key explanatory variables. However, separate models were preferred in this study to allow for more context-specific interpretation of determinants (71–78). This approach enables clearer identification of factors operating within each residential context, whilst acknowledging the limitations of formal statistical comparison.

## 4 Results

### 4.1 Descriptive statistics

The study found that 84.7% of women residing in rural areas and 80.4% of women residing in urban areas had been subjected to controlling behaviour by their spouse/partner in the past year at the time of the survey. Findings on factors associated with women’s exposure to controlling behaviour by place of residence in Türkiye are presented in **Table 1**.

**Table 1:** Descriptive characteristics of women and factors associated with exposure to controlling behaviour by partners by place of residence in Türkiye.

Variable	Whole model		Rural		Urban	
	n	%	n	%	n	%
Generation						
1946-1964	1,163	18.0	476	23.2	687	15.6
1965-1980	2,729	42.3	857	41.7	1,872	42.5
1981-1995	2,368	36.7	654	31.8	1,714	38.9
1996 and later	198	3.1	67	3.3	131	3.0
Education Level						
Illiterate	1,241	19.2	573	27.9	668	15.2
Primary school	2,907	45.0	1,067	51.9	1,840	41.8
Secondary school	879	13.6	233	11.3	646	14.7
High school	916	14.2	135	6.6	781	17.7
University	515	8.0	46	2.2	469	10.6
Women’s greater contribution to household income						
No	6,136	95.0	1,978	96.3	4,158	94.4
Yes	322	5.0	76	3.7	246	5.6
Suicidal thoughts						
No	5,263	81.5	1,716	83.5	3,547	80.5
Yes	1,195	18.5	338	16.5	857	19.5
Previous pregnancy						
No	728	11.3	197	9.6	531	12.1
Yes	5,730	88.7	1,857	90.4	3,873	87.9
Spouse/partner’s level of education						
Illiterate	378	5.9	156	7.6	222	5.0
Primary school	2,738	42.4	1,166	56.8	1,572	35.7
Secondary school	1,050	16.3	308	15.0	742	16.8
High school	1,456	22.5	319	15.5	1,137	25.8
University	836	12.9	105	5.1	731	16.6
The situation where the spouse/partner engages in a physical altercation with the man involving physical violence						
No	5,803	89.9	1,886	91.8	3,917	88.9
Yes	655	10.1	168	8.2	487	11.1
Whether or not it is acceptable for a man to beat his wife						
No	3,940	61.0	1,049	51.1	2,891	65.6
Yes	2,518	39.0	1,005	48.9	1,513	34.4
Being subjected to economic violence by one’s spouse/partner						
No	4,704	72.8	1,615	78.6	3,089	70.1
Yes	1,754	27.2	439	21.4	1,315	29.9
Being subjected to emotional abuse by one’s spouse/partner						
No	3,795	58.8	1,249	60.8	2,546	57.8
Yes	2,663	41.2	805	39.2	1,858	42.2
Being subjected to physical violence by one’s spouse/partner						
No	4,329	67.0	1,340	65.2	2,989	67.9
Yes	2,129	33.0	714	34.8	1,415	32.1
Fear of spouse/partner						
No	5,388	83.4	1,687	82.1	3,701	84.0
Yes	1,070	16.6	367	17.9	703	16.0
Knowledge of protective measures against violence						
No	414	6.4	234	11.4	180	4.1
Yes	6,044	93.6	1,820	88.6	4,224	95.9
Situation of physical violence experienced by spouse/partner from their own family						
No	5,192	80.4	1,689	82.2	3,503	79.5
Yes	1,266	19.6	365	17.8	901	20.5
Household size						
3 and under	2,325	36.0	671	32.7	1,654	37.6
4-5	2,898	44.9	801	39.0	2,097	47.6
6 and above	1,235	19.1	582	28.3	653	14.8
Place of residence						
Rural	2,054	31.8				
Urban	4,404	68.2				

### 4.2 Model estimation

In the study, multicollinearity amongst the independent variables to be included in the binary logistic regression model was assessed. It is stated that variance inflation factor (VIF) values of 5 and above indicate moderate multicollinearity, whilst values of 10 and above indicate high multicollinearity. In the present study, no independent variables exhibit multicollinearity (**Appendix 1**).

The estimated results of the binary logistic regression model are presented in **Table 2**. In the model estimated for all women participating in the study, women’s place of residence is associated with exposure to controlling behaviour.

**Table 2:** Estimated model results for factors associated with women’s exposure to controlling behaviour according to their place of residence.

Variables	Whole model		Rural		Urban	
	β	Std. Error	β	Std. Error	β	Std. Error
**Constant**	0.753^a^	0.256	0.527	0.443	0.463	0.333
Generation (reference: 1946-1964)						
1965-1980	0.102	0.116	-0.011	0.196	0.140	0.139
1981-1995	0.721^a^	0.130	0.574^b^	0.250	0.762^a^	0.152
1996 and later	1.178^a^	0.344	0.869	0.557	1.285^a^	0.418
Level of education (reference: illiterate)						
Primary school	-0.180	0.130	-0.294	0.184	-0.132	0.170
Secondary school	-0.286	0.179	-0.030	0.339	-0.303	0.214
High school	-0.385^b^	0.173	-0.323	0.337	-0.352^c^	0.207
University	-0.651^a^	0.204	-1.169^b^	0.478	-0.588^b^	0.236
The woman’s greater contribution to household income (reference: no)						
Yes	-0.274 ^c^	0.165	-0.003	0.377	-0.323^c^	0.185
Suicidal thoughts (reference: no)						
Yes	0.411^a^	0.130	0.337	0.239	0.433^a^	0.149
Previous pregnancy (reference: no)						
Yes	-0.533^a^	0.145	0.055	0.302	-0.642^a^	0.164
Spouse/partner’s level of education (reference: primary school)						
Illiterate	-0.583^a^	0.192	-0.343	0.295	-0.687^a^	0.242
Secondary school	-0.152	-0.312	0.227	-0.312	-0.119	0.153
High school	-0.101	-0.052	0.216	-0.052	-0.127	0.138
University	-0.054	0.114	0.339	0.114	-0.077	0.162
The situation where the spouse/partner engages in a physical altercation with a man involving physical violence (reference: no)						
Yes	0.409^b^	0.186	0.285	0.366	0.420^b^	0.211
Whether or not it is acceptable for a man to beat his wife (reference: no)						
Yes	0.812^a^	0.099	0.789^a^	0.152	0.837^a^	0.124
Exposure to economic violence by spouse/partner (reference: no)						
Yes	0.815^a^	0.118	0.766^a^	0.244	0.825^a^	0.133
Exposure to emotional abuse by spouse/partner (reference: no)						
Yes	0.459^a^	0.102	0.742^a^	0.192	0.392^a^	0.117
Experience of physical violence from spouse/partner (reference: no)						
Yes	0.493^a^	0.117	0.104	0.184	0.616^a^	0.142
Fear of spouse/partner (reference: no)						
Yes	0.509^a^	0.147	0.170	0.242	0.624^a^	0.178
Knowledge of protective measures against violence (reference: no)						
Yes	0.680^a^	0.151	0.558^a^	0.207	0.800^a^	0.211
Whether the spouse/partner has experienced physical violence from their own family (reference: no)						
Yes	0.207^c^	0.118	0.427^c^	0.222	0.153	0.137
Household size (reference: 6 and above)						
3 and under	-0.179	0.135	-0.363^c^	0.205	-0.129	0.173
4-5	-0.238^c^	0.126	-0.177	0.184	-0.242	0.163
Place of residence (reference: rural)						
Urban	-0.189^b^	0.094				

^a^ p<.01; ^b^p<.05; ^c^p<.10. Bold values: Independent variables.

According to **Table 2**, in the estimated model for women living in rural areas, generation (1981-1995), level of education (university), whether or not they consider it acceptable for a man to beat his wife, exposure to economic violence by a spouse/partner, exposure to emotional violence by a spouse/partner, knowledge of measures to prevent violence, physical violence by a spouse/partner’s own family, and household size (3-6) are significant. In the estimated model for women living in cities, generation (1981-1995, 1996 and later), educational level (secondary school, university), the woman’s greater contribution to household income, suicidal thoughts, previous pregnancy, spouse/partner’s educational level (illiterate), spouse/partner engaging in physical violence with the man, agreeing or disagreeing with the man beating his wife, exposure to economic violence from spouse/partner, exposure to emotional violence from spouse/partner, exposure to physical violence from spouse/partner, fear of spouse/partner, and knowledge of preventive measures against violence.

The marginal effects of factors associated with women’s exposure to controlling behaviour, by place of residence, are presented in **Table 3**.

**Table 3:** Marginal effects of factors associated with women’s exposure to controlling behaviour according to their place of residence.

Variables	Whole Model		Rural		Urban	
	ME	Std. Error	ME	Std. Error	ME	Std. Error
Generation (reference: 1946-1964)						
1965-1980	0.024	0.027	-0.002	0.037	0.034	0.035
1981-1995	0.136^a^	0.027	0.087^b^	0.039	0.153^a^	0.034
1996 and later	0.191^a^	0.041	0.119^b^	0.061	0.217^a^	0.051
Level of education (reference: illiterate)						
Primary school	-0.031	0.021	-0.045^c^	0.027	-0.023	0.029
Secondary school	-0.051	0.032	-0.004	0.047	-0.057	0.040
High school	-0.071^b^	0.032	-0.050	0.056	-0.067^c^	0.038
University	-0.132^a^	0.044	-0.253^c^	0.139	-0.122^b^	0.050
The woman’s greater contribution to household income (reference: no)						
Yes	-0.057	0.038	-0.001	0.062	-0.071	0.045
Suicidal thoughts (reference: no)						
Yes	0.071^a^	0.020	0.050	0.032	0.077^a^	0.024
Previous pregnancy (reference: no)						
Yes	-0.088^a^	0.021	0.009	0.051	-0.108^a^	0.023
Spouse/partner’s level of education (reference: primary school)						
Illiterate	-0.130^c^	0.050	-0.061	0.058	-0.164^b^	0.069
Secondary school	-0.029	0.025	-0.055	0.043	-0.023	0.030
High school	-0.019	0.023	0.008	0.033	-0.025	0.027
University	-0.010	0.027	0.017	0.049	-0.015	0.031
The situation where the spouse/partner engages in a physical altercation with a man involving physical violence (reference: no)						
Yes	0.069^b^	0.027	0.042	0.049	0.074^b^	0.032
Whether or not it is acceptable for a man to beat his wife (reference: no)						
Yes	0.138^a^	0.015	0.124^a^	0.024	0.145^a^	0.019
Exposure to economic violence by spouse/partner (reference: no)						
Yes	0.131^a^	0.016	0.102^a^	0.026	0.140^a^	0.019
Exposure to emotional abuse by spouse/partner (reference: no)						
Yes	0.083^a^	0.017	0.108^a^	0.025	0.075^a^	0.021
Experience of physical violence from spouse/partner (reference: no)						
Yes	0.086^a^	0.019	0.017	0.029	0.109^a^	0.022
**Fear of spouse/partner (reference: no)**						
Yes	0.085^a^	0.021	0.026	0.036	0.105^a^	0.025
Knowledge of protective measures against violence (reference: no)						
Yes	0.161^a^	0.043	0.106^b^	0.046	0.206^a^	0.067
Whether the spouse/partner has experienced physical violence from their own family (reference: no)						
Yes	0.038^c^	0.021	0.062^b^	0.029	0.030	0.025
Household size (reference: 6 and above)						
3 and under	-0.032	0.024	-0.058^c^	0.033	-0.024^c^	0.031
4-5	-0.044^b^	0.022	-0.026	0.027	-0.047	0.030
Place of residence (reference: rural) .						
Urban	-0.035^b^	0.017				

^a^ p<.01; ^b^p<.05; ^c^p<.10. Bold values: Independent variables.

Women living in rural areas born between 1981 and 1995 and those born in 1996 and later are 8.7% and 11.9% more likely, respectively, to experience controlling behaviour than women born between 1946 and 1964 (reference group). Women with primary and university education levels are 4.5% and 25.3% less likely to be subjected to controlling behaviour than women who cannot read or write. Women who approve of their husbands beating them are 12.4% more likely to be subjected to controlling behaviour than others. Women who experience economic and emotional violence from their spouse/partner are 10.2% and 10.8% more likely to experience controlling behaviour than women who do not experience these types of violence. Women who are aware of preventive measures against violence are 10.6% more likely to experience controlling behaviour than others. Women whose spouse/partner has been subjected to physical violence by their own family are 6.2% more likely to be subjected to controlling behaviour than others. Women in households of 3 or fewer members are 5.8% more likely to be subjected to controlling behaviour than women in households of 6 or more members.

Women living in urban areas born between 1981 and 1995 and those born in 1996 and later are 15.3% and 21.7% more likely to experience controlling behaviour than women born between 1946 and 1964, respectively. Women with secondary and university education are 6.7% and 12.2% less likely to be subjected to controlling behaviour than women who cannot read or write. Women who have suicidal thoughts are 7.7% more likely to be subjected to controlling behaviour than women who do not have suicidal thoughts. Women who have been pregnant before are 10.8% less likely to be subjected to controlling behaviour than others. Women whose spouse/partner is illiterate are 16.4% less likely to be subjected to controlling behaviour than those whose spouse/partner has completed primary school. Women whose spouse/partner physically fights with men are 7.4% more likely to be subjected to controlling behaviour than others. Women who approve of their husband beating his wife are 14.5% more likely to be subjected to controlling behaviour than others. Women who experience economic, emotional, and physical violence from their spouse/partner are 14.0%, 7.5%, and 10.9% more likely to experience controlling behaviour than women who do not experience these types of violence. Women who fear their spouse/partner are 10.5% more likely to experience controlling behaviour than others. Women who are aware of preventive measures against violence are 20.6% more likely to experience controlling behaviour than others. Women in households of three or fewer members are 2.4% less likely to experience controlling behaviour than women in households of six or more members.

## 5 Discussion

Women’s empowerment has been widely recognised as a key factor influencing women’s exposure to intimate partner violence, including controlling behaviours. Increased levels of education, economic participation, and decision-making autonomy can enhance women’s bargaining power within relationships and reduce dependence on male partners, thereby potentially lowering the risk of controlling behaviours (12, 13). However, the relationship between empowerment and controlling behaviour is not always linear. The literature highlights a “backlash effect,” whereby shifts in traditional gender roles and increased female autonomy may be perceived as a threat to established power hierarchies, leading some male partners to intensify controlling and coercive behaviours in an effort to reassert dominance (14, 15). This dual dynamic suggests that women’s empowerment operates within broader socio-cultural and structural contexts, particularly gender norms and patriarchal values, which shape how empowerment translates into either protection or increased risk. Therefore, examining empowerment-related variables alongside relational and contextual factors is essential for understanding the complex mechanisms underlying controlling behaviours in intimate partnerships.

Controlling behaviour towards women by their partners is recognised in international literature as a significant component of psychological violence and has serious effects on women’s physical, psychological and social health (79, 80). Research indicates that multiple factors at the individual, relationship and societal levels play a role in the emergence of these behaviours. In particular, differences between rural and urban settlements emerge as a significant variable affecting women’s likelihood of being subjected to controlling behaviour. At the same time, educational status, economic independence and certain characteristics of partners also influence women’s exposure to controlling behaviour by their partners.

There are also social and cultural differences between rural and urban areas. These differences can lead to different behaviours and practices. To identify these differences, this study used binary logistic regression to examine factors affecting women’s exposure to controlling behaviour by place of residence in Türkiye. It was found that the significance and effect of the variables on women’s exposure to controlling behaviour varied by place of residence.

In rural areas, the variables of generation, level of education, whether or not the man beating his wife is considered acceptable, exposure to economic violence by the spouse/partner, exposure to emotional violence by the spouse/partner, knowledge of measures to prevent violence, and whether the spouse/partner has experienced physical violence from their own family. Household size is associated with women’s exposure to controlling behaviour.

In urban areas, age, educational level, the woman’s greater contribution to household income, suicidal thoughts, previous pregnancies, the spouse/partner’s educational level, the spouse/partner engaging in physical violence against the man, whether or not the man beating his wife is considered acceptable, exposure to economic violence by the spouse/partner, exposure to emotional violence by the spouse/partner, exposure to physical violence by the spouse/partner, fear of the spouse/partner, and knowledge of measures to prevent violence are associated with women’s exposure to controlling behaviour.

According to the current study, women living in cities are less likely to be subjected to controlling behaviour by their partners than those living in rural areas. Similar to this research’s findings, a study conducted in Nigeria found that women living in rural areas are more likely to be subjected to controlling behaviour by their partners than those living in cities (67). Several reasons can explain this situation. The first is that women in cities have higher levels of education. This enables women to recognise controlling behaviour and develop resistance (67). Another reason is that women living in cities are less dependent on their partners because they are more likely to have income-generating jobs, which leads them to reject controlling behaviour (12). Stronger access for women in the city to law enforcement, legal mechanisms and health services compels their partners to reduce controlling behaviour (81, 82).

According to the findings of the study, women born in Generation Y and Z (1981-1995, 1996 onwards) were found to be more likely to be subjected to controlling behaviour by their partners than women born in the Baby Boom Generation (1946-1964). It is thought that women from Generations Y and Z, having been raised within more individualistic and egalitarian social norms, are more likely to notice controlling behaviour from their partners and to be subjected to it (83). While studies examining the likelihood of women being subjected to controlling behaviour by their partners about age exist in the literature, no study examining the likelihood of women being subjected to controlling behaviour by their partners about generational differences has been found. The present study contributes to the literature by examining the relationship between these two variables.

The study found that women with secondary and tertiary education levels were less likely to be subjected to controlling behaviour than illiterate women. Similarly, a study conducted in Ankara, Türkiye, revealed an inverse relationship between women’s educational levels and the likelihood of being subjected to controlling behaviour by their partners (84). Another study conducted in Delhi, India, also found that as women’s educational levels decrease, the likelihood of them being subjected to controlling behaviour by their partners increases (85). The common finding of these studies is that as women’s educational level decreases, their likelihood of being subjected to controlling behaviour decreases. Several reasons can explain this situation. As women’s educational level increases, their cognitive skills, employment opportunities, and professional status will expand, leading to an increase in women’s personal resources (86, 87). In contrast, these personal resources will increase women’s potential to leave abusive or unhappy relationships by reducing their economic dependence on their partners (88).

The study found that women who had suicidal thoughts were subjected to more controlling behaviour than women who did not have suicidal thoughts. A study conducted in Nepal revealed that women who were subjected to controlling behaviour by their partners were approximately twice as likely to have suicidal thoughts (57). As seen, the effect of controlling behaviour on suicidal ideation has been investigated in the literature; however, no study has been found that examines the effect of women’s suicidal ideation on their exposure to controlling behaviour by their partners, as included in the present study. The current study contributes to the literature by examining this effect as well.

According to the current study, it has been found that women who have been pregnant before are less likely to be subjected to controlling behaviour than women who have never been pregnant. Although no studies have been found that examine the relationship between these two variables in the same way, other studies have investigated it similarly. The findings of these studies differ from those of the current study. It has been determined that women are more likely to be subjected to controlling behaviour during their fertile period than during their non-fertile period (89). Cultural differences may explain these varying results. The present study was conducted across Türkiye, where motherhood, pregnancy, and having a baby are considered more sacred than in many other societies, and a woman’s value is largely associated with motherhood (90, 91). Again, in Turkish society, women are seen as figures who need to be “protected” during their childbearing years (92). However, it has been noted that during pregnancy, men’s behaviour, despite their patriarchal attitudes, is influenced by social roles, leading them to adopt a more sensitive approach towards their partners (93).

It has been found that women who consider it acceptable for men to beat their wives are subjected to more controlling behaviour by their partners. Parallel to this finding, according to the results of a study conducted in India, it has been observed that women who attribute violence to justifiable reasons are subjected to more controlling behaviour by their partners (85). Another study conducted in Nigeria also found that women who legitimise their husbands’ beating them are subjected to more controlling behaviour by their partners (53). Women’s acceptance or legitimisation of controlling behaviour by their partners is based on several key reasons. The first of these reasons is that women normalise violence or controlling behaviour by their partners through cultural norms or a perception of fate. In this case, whilst men reinforce their control within the relationship, women also increase their likelihood of being subjected to it (94). Another reason for the controlling justification that women are subjected to by their partners is that what begins as jealousy, surveillance, or decision-making is seen as love, protection, or a right (95). In addition to these reasons, some women view violence or control as a means of maintaining order within the relationship or preserving the male’s dominance. Therefore, they tend to be subjected to controlling behaviours without offering any resistance (96).

When the educational background of partners was examined, it was determined that women whose spouses/partners were illiterate were less likely to experience controlling behaviour compared to women whose partners had only completed primary school. Parallel to this finding, another study conducted in Türkiye found that women whose close partners were illiterate or lacked a diploma were less likely to experience controlling behaviour from their partners than women whose close partners had only completed primary school (18). However, according to findings from another study, there is no significant difference in the likelihood of experiencing controlling behaviour between women whose spouses/partners have completed middle school, high school, or university education and women whose partners have only completed primary school. This finding also indicates that there is no significant relationship between a spouse’s education level and the likelihood of exhibiting controlling behaviour (97).

According to the study’s findings, women whose husbands/partners engage in physically violent fights are more likely to experience controlling behaviours than women whose partners do not engage in physically violent fights. Parallel to this finding, another study conducted in Türkiye indicates that a woman’s close partner engaging in a physically violent fight with another man increases the likelihood of her experiencing controlling behaviours (18). A significant number of men who are violent or controlling towards their partners view the physical violence they inflict on other men as a form of control and a display of power (31). This mindset undoubtedly explains these men’s tendency to exhibit more controlling behaviour towards their wives.

The study found that women who experienced economic violence from their spouse/partner were more likely to experience controlling behaviour from their partners compared to women who did not experience this type of violence. Economic violence tends to occur alongside other forms of intimate partner violence. It is a strong indicator, particularly associated with controlling behaviour, including economic restriction, the man’s unilateral control over economic decisions, etc. (98). Partner behaviours that involve economic restrictions are often accompanied by limitations on the woman’s social relationships and on her control over decision-making (99). However, a male partner’s sole control over economic decisions is one of the most common ways to keep women under dominance and control, both psychologically and behaviourally (22).

According to current research findings, women who experience emotional abuse from their spouse/partner are more likely to experience controlling behaviour from their partners compared to women who do not experience this type of abuse. Similarly, a study conducted in North America concluded that there is a correlation between emotional abuse perpetrated by partners and the likelihood of experiencing controlling behaviour from their partners (23). In their study conducted in Canada, Ansara and Hindin (100) found a correlation between women’s experiences of emotional abuse and their experiences of controlling behaviour from their partners. Emotional abuse is defined as a primary form of control used by the male partner to maintain or reinforce his power balance in the relationship (101). This type of violence leads to controlling behaviours towards women by making them feel guilty, humiliating them, ignoring them, and interfering in their decision-making processes (25).

In the current study, women who experience physical violence from their spouse/partner are more likely to experience controlling behaviour from their partners compared to women who do not experience this type of violence. Findings from a study conducted in the USA showed that women who experience physical violence are significantly more likely to experience controlling behaviour from their partners, which is similar to the findings of the current research (102). Another study found a correlation between women experiencing physical violence and being subjected to controlling behaviour (103). The findings of a multi-country study spanning different continents, including Bangladesh, Brazil, Ethiopia, Japan, Kenya, Namibia, Peru, Serbia, Thailand, and Samoa, also parallel the findings of the current study, revealing that the presence of physical violence increases the likelihood of exposure to controlling behaviours (104). When women experience physical violence, it is often accompanied by their partners exhibiting controlling behaviour that affects their social lives, economic resources, and daily decisions (31). At the same time, physical violence is part of controlling behaviours aimed at directing a woman’s behaviour and creating dependency (1).

The study revealed that women who fear their spouse/partner are more likely to experience controlling behaviour from their partners. Parallel to these research findings, a study conducted in both urban and rural areas of South Africa indicated that women who fear their partners are more likely to experience controlling behaviours such as social isolation and restricted freedom of movement (25). Another study also found that women who fear their partners are more likely to be subjected to controlling social and behavioural behaviours (105). Women’s fear of their partners perpetuates a cycle involving violence and controlling behaviour. Fear prevents women from acting independently, reinforcing controlling behaviours (7).

Recent research has found that women who are aware of protective orders to prevent violence are more likely to experience controlling behaviour from their partners than women who are not. Similar findings from other studies in the US indicate that in some relationships, women’s knowledge of their legal rights and protective orders can lead to increased controlling behaviour from male partners (59). When women become aware of legal measures and their rights to prevent violence, some male partners perceive this as a threat to their power and control mechanisms and increase their behavioural control (106).

Women whose spouses/partners have experienced physical violence from their own families are more likely to experience controlling behaviour than women whose spouses/partners have not experienced physical violence from their own families. Similar to the findings of the current research, a study conducted in the Basque Country of Spain revealed that having experienced physical violence from a partner’s family played a significant role in the likelihood of developing controlling and oppressive behaviours towards one’s partner in adulthood (107). According to another study conducted in the US, experiencing physical violence from a partner’s own family is a significant factor that increases women’s risk of experiencing violence and controlling behaviour from their partners (108). According to Widom and Maxfield (109), men who experienced physical violence in childhood are at a higher risk of developing controlling and oppressive behaviours towards their partners later in life. Physical violence experienced by a partner within their own family can emerge as a determinant of power and control behaviours in future romantic relationships, causing the cycle of violence and control to continue across generations (110).

The current research indicates that women in households of 4–5 people are less likely to experience controlling behaviour than those in households of 6 or more people. No studies investigating the relationship between these two variables have been found. However, a study that yielded similar findings concluded that in Samoa, violence against women by their partners increased in larger families, and partners’ attitudes towards women also worsened (60). Women living in larger households are more likely to observe and interfere with their partners’ behaviour, leading to increased control and dominant behaviour on the part of their partners. Household size and family structure are determinants of the controlling behaviour a woman experiences from her partner; in extended families, the male partner’s authority is more emphasised, and controlling behaviours intensify (111).

## 6 Conclusion

Women who experience controlling behaviour from their partners experience negative impacts on their psychological, social, and economic lives. It is clear that controlling behaviour increases stress, anxiety, and depression levels in women; leads to isolation by limiting their social relationships; and restricts their economic independence. Controlling behaviour is influenced not only by individual relationship dynamics but also by societal, economic, and cultural factors. In other words, women’s exposure to controlling behaviour from their partners is shaped by gender norms, patriarchal values, and cultural expectations. For example, in rural areas, traditional gender roles restrict women’s economic and social mobility, making controlling behaviour from partners more visible and prevalent.

Controlling behaviours are considered a part of emotional abuse. Therefore, given the significant mental health consequences associated with emotional abuse, it is concerning that this type of violence often receives less attention, is not sufficiently studied, and is not adequately recognised. For example, the World Health Organisation’s global, regional, and national prevalence estimates primarily focus on physical and sexual violence. Without acknowledging and measuring emotional abuse and controlling behaviours, the health system cannot adapt to meet the needs of these women. Furthermore, this surveillance may reduce their likelihood of seeking help and thereby mitigate overall health outcomes. Therefore, strengthening surveillance systems to monitor violence is crucial and enables timely interventions (57).

The study shows that the significance and impact of variables in experiencing controlling behaviour from partners differ across women’s places of residence. The study’s findings include identifying factors specific to rural and urban areas. This study contributes to the literature by showing that the factors shaping women’s experience of controlling behaviour vary by place of residence.

In light of the study’s findings, it is suggested that policies and interventions aimed at preventing women from experiencing controlling behaviour from their partners should be addressed not only at the individual level but also at the societal and structural levels. Furthermore, structural factors such as geographical location differences and gender norms should be considered in research. The importance of programs that increase women’s education and economic independence, strengthen social support networks, and raise awareness should be emphasised, particularly in rural areas. For the entire society, regardless of region, the importance of structural measures such as increasing educational opportunities, supporting economic independence, and raising societal awareness should be highlighted through policies and interventions aimed at preventing and controlling the behaviour experienced by women.

Controlling behaviours exhibited by partners towards women are a form of violence against women and should also be considered a harbinger of physical violence. In this respect, the more controlling behaviours become recognisable and measurable, the more effective they will be in preventing violence. Therefore, the entire society should be made aware of controlling behaviours and guided by experts in the field.

### 6.1 Limitations of the study

There are several restrictions on this study. The data used in the study are secondary. The data collection already contains the variables needed for statistical analysis. Nevertheless, several factors could not be examined, such as homeownership status and occupation, which were not included in the data set. The women’s answers make up the information gathered about their exposure to controlling behaviour by their spouse/partner in the last year. As a result, the information gathered using this method could be skewed. The study’s data were collected 12 years ago. The Hacettepe University Institute of Population Studies provided data from the most recent National Research on Domestic Violence against Women in Türkiye, conducted in 2014. It is important to interpret the findings of this study within the temporal context of the data. The analysis is based on data collected in 2014, and social norms, policy frameworks, and awareness regarding violence against women in Türkiye may have evolved since then. Changes in legal regulations, increased public awareness, and expanded support mechanisms may have influenced both the prevalence and perception of controlling behaviours. Therefore, whilst the findings provide valuable insights, they should be interpreted with caution regarding their current applicability. In addition to estimating separate models for urban and rural samples, an alternative empirical strategy is a pooled model with interaction terms between place of residence and key explanatory variables. Such an approach would allow for formal statistical comparison of coefficients across groups and provide additional evidence on regional heterogeneity. However, separate models are preferred in this study to allow for more context-specific interpretation of the determinants of controlling behaviour. Lastly, the findings should be interpreted as associations rather than causal relationships, given the cross-sectional design of the data.

## Data Availability

The data underlying this study is subject to third-party restrictions by the Turkish Statistical Institute. Data are available from the Turkish Statistical Institute (bilgi@tuik.gov.tr) for researchers who meet the criteria for access to confidential data. Further inquiries can be directed to the corresponding author/s.

## Appendix

**Table:** Appendix 1. VIF values for all models.

Variables	Whole Model		Rural		Urban	
	VIF	1/VIF	VIF	1/VIF	VIF	1/VIF
Generation (reference: 1946-1964)						
1965-1980	2.25	0.444	2.02	0.494	2.41	0.415
1981-1995	2.52	0.397	2.44	0.411	2.63	0.381
1996 and later	1.71	0.586	1.75	0.570	1.70	0.589
Level of education (reference: illiterate)						
Primary school	2.15	0.464	1.63	0.615	2.53	0.395
Secondary school	1.99	0.503	1.76	0.567	2.17	0.460
High school	2.13	0.469	1.51	0.662	2.47	0.404
University	2.18	0.460	1.45	0.690	2.50	0.401
The woman’s greater contribution to household income (reference: no)						
Yes	1.05	0.953	1.03	0.967	1.06	0.946
Suicidal thoughts (reference: no)						
Yes	1.14	0.881	1.11	0.900	1.15	0.868
Previous pregnancy (reference: no)						
Yes	1.41	0.707	1.49	0.670	1.39	0.718
Spouse/partner’s level of education (reference: primary school)						
Illiterate	1.15	0.869	1.13	0.885	1.17	0.856
Secondary school	1.30	0.768	1.22	0.823	1.36	0.738
High school	1.47	0.679	1.31	0.762	1.53	0.652
University	1.80	0.556	1.36	0.734	1.90	0.525
The situation where the spouse/partner engages in a physical altercation with a man involving physical violence (reference: no)						
Yes	1.10	0.905	1.11	0.900	1.10	0.906
Whether or not it is acceptable for a man to beat his wife (reference: no)						
Yes	1.13	0.889	1.10	0.910	1.11	0.898
Exposure to economic violence by spouse/partner (reference: no)						
Yes	1.18	0.849	1.15	0.869	1.19	0.842
Exposure to emotional abuse by spouse/partner (reference: no)						
Yes	1.45	0.689	1.42	0.704	1.47	0.680
Exposure to physical violence by spouse/partner (reference: no)						
Yes	1.52	0.659	1.49	0.672	1.54	0.650
Fear of spouse/partner (reference: no)						
Yes	1.16	0.859	1.20	0.832	1.15	0.869
Yes	1.06	0.941	1.07	0.938	1.03	0.967
Whether the spouse/partner has experienced physical violence from their own family (reference: no)						
Yes	1.10	0.910	1.11	0.903	1.10	0.910
Household size (reference: 6 and above)						
3 and under	2.22	0.450	1.88	0.532	2.55	0.393
4-5	1.98	0.506	1.53	0.652	2.32	0.431
Place of residence (reference: rural)						
Urban	1.13	0.883				
